# Message hidden in α-helices—toward a better understanding of plant ABCG transporters’ multispecificity

**DOI:** 10.1093/plphys/kiaf146

**Published:** 2025-04-12

**Authors:** Wanda Biała-Leonhard, Aleksandra Bigos, Jan Brezovsky, Michał Jasiński

**Affiliations:** Department of Plant Molecular Physiology, Institute of Bioorganic Chemistry, Polish Academy of Sciences, 61-704 Poznan, Poland; Faculty of Biology, Department of Gene Expression, Laboratory of Biomolecular Interactions and Transport, Institute of Molecular Biology and Biotechnology, Adam Mickiewicz University, 61-614 Poznan, Poland; International Institute of Molecular and Cell Biology, 02-109 Warsaw, Poland; Faculty of Biology, Department of Gene Expression, Laboratory of Biomolecular Interactions and Transport, Institute of Molecular Biology and Biotechnology, Adam Mickiewicz University, 61-614 Poznan, Poland; International Institute of Molecular and Cell Biology, 02-109 Warsaw, Poland; Department of Plant Molecular Physiology, Institute of Bioorganic Chemistry, Polish Academy of Sciences, 61-704 Poznan, Poland

##  

ATP-binding cassette (ABC) transporters are ubiquitous in all organisms and constitute one of the largest protein families. The substantial expansion of this family in plants coincided with the emergence of fundamental novelties that facilitated successful adaptation to a sessile lifestyle on land. It also resulted in selectivity and multispecificity toward endogenous molecules observed for certain ABC transporters. Understanding the molecular determinants behind this intriguing feature remains an ongoing challenge for the functional characterization of these proteins. This review synthesizes current achievements and methodologies that enhance our mechanistic understanding of how ABCG transporters, which are particularly numerous in land plants, specifically recognize and transport endogenous compounds. We examine in silico modeling and the recent advancements in the structural biology of ABCGs. Furthermore, we highlight internal and external factors that potentially influence the substrate selectivity of those proteins. Ultimately, this review contributes to rationalizing our current capacity to fully understand how plants orchestrate membrane transport fulfilled by these proteins.

## Plant ABCG transporters—from detoxifiers to multispecific distributors

ABC transporters are integral membrane-bound primary active pumps that are ubiquitously present in prokaryotes and eukaryotes. The structural framework of functional full-size eukaryotic ABC transporters consists of a single polypeptide containing 2 transmembrane domains (TMDs) that form the translocation pathway across the membrane bilayer; and 2 nucleotide-binding domains (NBDs), where ATP binding and hydrolysis occur (Rees et al. 2009). Additionally, half-size ABC proteins exist and consist of a single TMD and a single NBD, which can assemble into hetero- or homodimers to function. Also, soluble proteins comprising only 2 NBDs as well as non-intrinsic ABC proteins, containing only 1 domain (TMD or NBD), can be found in plants. The ABC family is divided into 9 subfamilies (from ABCA to ABCI) according to their domain organization and their phylogenetic relationships. Every subfamily but ABCH is represented in plants (Verrier et al. 2008; Hwang et al. 2016). The ATP binding and hydrolysis are likely to facilitate the conformational transition during the transport cycle. NBDs are well conserved, while TMDs are polyphyletic and exhibit distinct folds (Thomas et al. 2020). For instance, in proteins from ABCB subfamily α-helices forming TMD display so-called domain swap arrangement, while in ABCG subfamily members, instead of rotation of individual α-helices, the entire TMD rotates as a solid body during the transport of the substrate (Ford and Beis 2019).

Compared with other organisms, plant ABCs are particularly numerous (Hwang et al. 2016). It is proposed that the noticeable expansion, especially of the ABCB and ABCG subfamilies in land plants, was crucial for adaptation to the new environment as well as unique developmental processes (for review, see Hwang et al. 2016; Banasiak and Jasiński 2022). Initial research regarding plant full-size ABCG transporters primarily focused on their role in detoxification and pathogen defense through the transport of xenobiotics and specialized metabolites (Do et al. 2021). Plant half-size ABCG proteins were initially considered as lipid transporters by their structural similarity to human homologs (Lefèvre and Boutry 2018). However, the context of detoxification evolved, and we have gained insights into the precise tuning of plant ABCG proteins not only from a substrate selectivity perspective but also as essential components for organ growth, plant nutrition, development, response to abiotic stresses, and the interaction of the plant with its environment (Do et al. 2018, 2021). The extensive chemical diversification of specialized metabolism and the spatially separated action of phytohormones created evolutionary pressure on ABC transporters, leading to selective translocation of endogenous molecules. This selectivity issue together with biological meaning is well documented for several ABCG transporters and emerges as an important aspect of their action. For instance, ABCG29 from *Arabidopsis thaliana* is translocating 1 of 3 main lignin precursors—*p*-coumaryl alcohol, while 2 other monolignols, such as coniferyl alcohol and sinapyl alcohol, are not transported despite their chemical/structural similarity (Miao and Liu 2010; Alejandro et al. 2012). Then, *Catharantus roseus* ABCG1/TPT2 was shown to specifically transport catharanthine compared with other monoterpenoid indole alkaloids such as vindoline, tabersonine, or strictosidine (Yu and De Luca 2013). Similarly, ABCG1/TPT2 from *Vinca minor* appears to have a preference toward vincamine but not to vincadifformine (Demessie et al. 2017). Also, *Artemisia annua* ABCG3/PDR3 transports β-caryophyllene and discriminates other terpenoids such as β-farnesene and germacrene D (Fu et al. 2017). Furthermore, a full-size AtABCG40/PDR12 abscisic acid (ABA) importer, and a half-size homodimer AtABCG25 ABA exporter highlight the selectivity of plant ABCG proteins. Both mentioned ABA transporters exhibit selectivity toward ABA enantiomers (S)-(+)-ABA and (R)-(−)-ABA (Kang et al. 2010; Kuromori et al. 2010; Huang et al. 2023).

**Figure 1.**
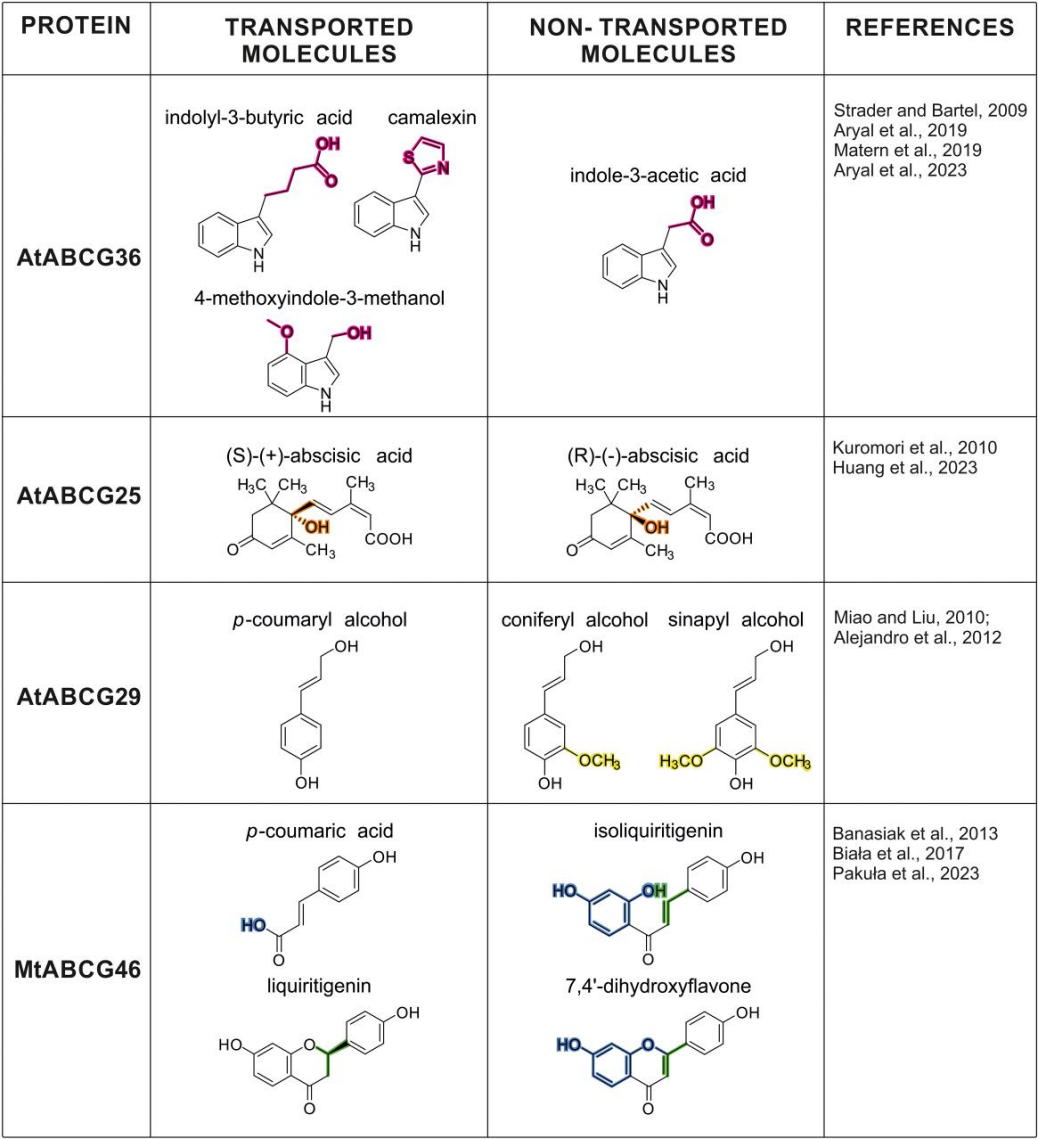
Comparison of ABCG's transported and nontransported molecules. Molecules tested in transport assays for chosen examples of plant ABCG proteins are presented as structural formulas. Differentiating elements within each group of molecules are highlighted in colors: AtABCG36—pink, AtABCG25—orange, AtABCG29—yellow, and MtABCG46—green and blue.

Recent findings regarding plant ABCG transporters have led to the concept of multispecificity, which is defined as the highly specific and selective translocation of a limited number of molecules. Importantly, the term plant multispecificity differs from commonly recognized substrate polyspecificity, where a single protein was able to nonselectively translocate a wide range of endogenous and exogenous compounds, described for—for example, yeast ABCGs and ABCBs involved in MDR (see Geisler 2024). An illustration of such a multispecific protein is, for example, *Nicotiana tabacum* ABCG1/PDR1, involved in constitutive defense against pathogens. NtABCG1 was shown to selectively translocate cyclic diterpenes sclareol, manool, and cembrane, while monoterpenes such as eucalyptol are discriminated against (Crouzet et al. 2013; Pierman et al. 2017). Further, ABCG37/PDR9 from Arabidopsis, which is engaged in the export of phytohormone indole-3-acetic acid (IAA) precursor, indolyl-3-butyric acid (IBA) (Růžička et al. 2010; Aryal et al. 2019), was also indirectly shown to be involved in the transport of scopoletin, a hydroxycoumarin that facilitates plant iron uptake from the soil (Fourcroy et al. 2014; Ziegler et al. 2017). Intriguingly, ABCG36/PDR8/PEN3, the closest homolog of AtABCG37, has even wider spectra, being involved in growth-defense decisions (Aryal et al. 2023). The ABCG36 is translocating the range of compounds such as signaling defense compound 4-methoxyindole-3-methanol (Matern et al. 2019), phytoalexin-camalexin (Aryal et al. 2023), and simultaneously a precursor of IAA phytohormone—IBA (Strader and Bartel 2009; Aryal et al. 2019). In legumes, an example of a multispecific transporter is ABCG46 from *Medicago truncatula*. This protein was shown to be required for efficient de novo biosynthesis of phenylpropanoid-derived phytoalexin medicarpin (Banasiak et al. 2013). Furthermore, MtABCG46 was characterized as a selective transporter of 2 structurally different medicarpin early precursors, named *p*-coumaric acid and liquiritigenin (Biała et al. 2017). Interestingly, both molecules translocated by MtABCG46 serve as important branch points in the phenylpropanoid pathway, with p-coumaric acid leading to distinct pathways for monolignols, coumarins, and (iso)flavonoids formation. Liquiritigenin is an immediate substrate for the biosynthesis of 5-deoxyflavonoids in Medicago related to signaling in symbiotic interactions (Zhang et al. 2009) and biosynthesis of 5-deoxyisoflavonoids associated with defense response (Naoumkina et al. 2010). Notably, structurally similar molecules originating from the same metabolic pathway (e.g. isoliquiritigenin or 7,4′-dihydroxyflavone) are not transported by MtABCG46 (Biała et al. 2017) (Fig. 1).

## Searching for molecular determinants behind the selectivity of plant ABCGs

The increasing number of described ABCG proteins as well as molecules translocated by them from different plant species allows for targeted application of phylogenetic comparison and clustering that may help in the challenging initial step, which is potential substrate assignment (Borghi et al. 2019; Banasiak and Jasiński 2022). The phylogeny may be further supported by tools such as Consurf (Celniker et al. 2013; Ashkenazy et al. 2016) and GREMLIN (Kamisetty et al. 2013; Ovchinnikov et al. 2014) dedicated to analyses of amino acid conservation and coevolution, respectively, to reveal potential coevolutionary connections and/or possible functional constraints. The latter approaches support the identification of both transporters and their relation with endogenous substrates. Recently, these tools were successfully applied also for mapping of residues such as F562 potentially involved in the selective transport of phenylpropanoids driven by MtABCG46 (Pakuła et al. 2023) or L704 that appeared to be a *Brassiceae* family-specific residue involved in observed discernment toward indolic compounds (Xia et al. 2024).

The structures of eukaryotic ABCG family members have begun to emerge in the last few years with the crystal structure of heterodimeric human ABCG5/G8 cholesterol exporter (Lee et al. 2016) and cryogenic electron microscopy (cryo-EM) derived structure of the homodimeric ABCG2 xenobiotic exporter (Taylor et al. 2017). Because of limited structural data for plant ABCs, in silico modeling is still an alternative choice while searching for molecular determinants behind the interaction of translocated molecules with transporters. Tools such as AlphaFold2 (AF2) enable 3-dimensional protein modeling based on amino acid sequences (Jumper et al. 2021) and, when combined with molecular dynamics (MD) simulations, can provide insights into protein dynamics and molecular interactions. However, the accuracy of AF2 depends on the availability of data for homologous proteins in databases, which is limited for certain protein families, also including transmembrane proteins (Jambrich et al. 2023). The reliability of AF2 for modeling transmembrane proteins has been extensively tested, including studies on the fold of the ABC protein superfamily (Hegedűs et al. 2022; Tordai et al. 2022). Moreover, AF2 is effective at predicting single stable conformations, mainly holo forms (Saldaño et al. 2022) as sequence-based predictions lack encoded information about conformational flexibility (Chakravarty et al. 2025). Hence, several approaches enabling the generation of alternative conformational states by manipulating data on homologous proteins were developed (Del Alamo et al. 2022). However, AF2 struggles with predictions of larger disordered regions (Stevens and He 2022) and potentially also when estimating the impact of point mutations or posttranslational modification on the protein conformation (Buel and Walters 2022; McBride et al. 2023), underscoring the need for critical evaluation of these predictions and the use of complementary methods. AlphaFold3 expands the application to the modeling structures of nucleic acids and biomolecular complexes, involving also small molecules and ions (Abramson et al. 2024). Nonetheless, recent use cases indicate that the novel architecture of AF3 still produced varying levels of accuracy, possibly also due to structural “hallucinations,” that again require careful consideration of delivered predictions (Desai et al. 2024; Kovalenko et al. 2024; Träger et al. 2024; Wee and Wei 2024).

Furthermore, studies using AF2 modeling of full-size ABCG transporters, including MtABCG46 (Pakuła et al. 2023) and AtABCG36 (Hegedűs et al. 2022; Xia et al. 2024), identified candidate residues potentially involved in substrate selectivity and multispecificity. To elucidate the role of those particular residues, models (presenting the typical arrangement of domains for full-size ABCG proteins) were further investigated by combining a wide range of computational tools and MD simulations. In the case of MtABCG46, exploration of TMDs by software like CAVER 3.0 together with TransportTools allowed for the identification of potential translocation pathways, an overview of the translocation path networks, and the positioning of crucial F562 within them (TMD helix 2) (Chovancova et al. 2012; Brezovsky et al. 2022; Pakuła et al. 2023). Furthermore, MD simulations and analyses of energetic profiles of the binding energy between the substrate(s) and MtABCG46 along the path by CaverDock (Vavra et al. 2019) were applied to explain disturbances of transport selectivity observed for F562-mutated variants in in vivo transport assays. The latter revealed that the substitution of F562 affects the bending and twisting angles of TMD helices, resulting in a reduction of space between helices and thus finally affecting the viability of paths for the access of particular molecules (Pakuła et al. 2023). In the model of AtABCG36, L704 was positioned in the TMD helix 5. Subsequently, this model was used in MD simulations, including well-tempered metadynamic simulations of substrate translocation to calculate corresponding free energy surface, supplemented with pulling simulations to investigate the role of extracellular gate in the process in detail. This allowed to show the differences in the free energy surface profiles around the central binding pocket. Also, pulling forces indicated that IBA and camalexin transport is controlled by various regions of the translocation path in AtABCG36. These observations genuinely supported complex biochemical analyses of various mutants, pointing to L704 as a key residue contributing to the observed AtABCG36 substrate selectivity (Xia et al. 2024). The mentioned examples demonstrate the usefulness of bioinformatic tools that supplement the functional characterization of plant ABCG proteins.

Recent advances in single-particle cryo-EM have not only delivered crucial information on the architecture of several ABC transporters but also allowed us to gain detailed mechanistic insights into their mode of action. Until recently, there was no experimental structure for plant ABCG transporters. The closest available ones were limited to a few of the aforementioned human half-size ABCG proteins and the structure of the first full-size ABCG transporter, PDR5, from *Saccharomyces cerevisiae* (Harris et al. 2021). The latter is a nonspecific transporter of a wide range of unrelated molecules, while its physiological substrate(s) are unknown (Golin and Schmitt 2023). Its structure was solved with single-particle cryo-EM, revealing the details of an ATP-driven conformational change, which mechanically forces drug translocation through an amphipathic channel (Harris et al. 2021). Recently, 3 independent groups published cryo-EM structures of homodimer ABCG25 from Arabidopsis (Huang et al. 2023; Ying et al. 2023; Xin et al. 2024). Detailed studies of inward-facing apo and ABA-bound conformations and outward-facing ATP-bound conformation depicted the architecture of AtABCG25 and how it recognizes ABA as well as allowed to trace the structural transitions during the ABA molecule translocation (Huang et al. 2023). Analysis of AtABCG25 revealed a cone-shaped, predominantly hydrophobic cavity formed by TMD helices 1, 2, and 5 from each monomer. Within this cavity, a single ABA molecule was positioned with its carboxylate tail oriented toward the cavity bottom and its ring group head facing the cytoplasmic side (Ying et al. 2023). Furthermore, characteristic and meaningful determinants were observed. The extracellular area of the cavity is sealed by a so-called “apoplastic gate” formed by Y565 (TMD helix 5a/5a′). Interestingly, the general structure of the protein in the apo state is very similar to the protein bound with an ABA molecule. The situation is changing drastically upon ATP binding. ATP forms intense interactions with residues from both NBDs, pulling them close together. Previously observed cavities disappear, and the transmembrane region is sealed from both sides. Also, the AtABCG25 has a “cytoplasmic gate” formed by F453 (TMD helix2/2′) (Huang et al. 2023; Ying et al. 2023). Very recently, a homodimer of another ABCG protein involved in the transport of the phytohormone jasmonic acid (JA), named AtABCG16, was structurally characterized (An et al. 2024). Apart from overall organization similar to AtABCG25, the structural analysis revealed the presence of additional extracellular domain between TMD helices 5 and 6 in each monomer and the unique structural feature—being the bifurcated translocation pathway. The latter is composed of 2 independent substrate entrances (formed by residues T460 and Y601) leading to substrate binding pockets, enabling simultaneous binding of 2 JA molecules (controlled by Y494), and further leading to shared apoplastic cavity closed by middle-TM gate formed by F608 and F609 (TMD helix5a/5a′) (An et al. 2024). The description of these structural determinants was possible due to the visualization of several bound states, highlighting the importance of a comprehensive understanding of structural rearrangements to the different functional state transitions.

## Intrinsic and exogenous factors shaping selectivity of plant ABCGs

**Figure 2.**
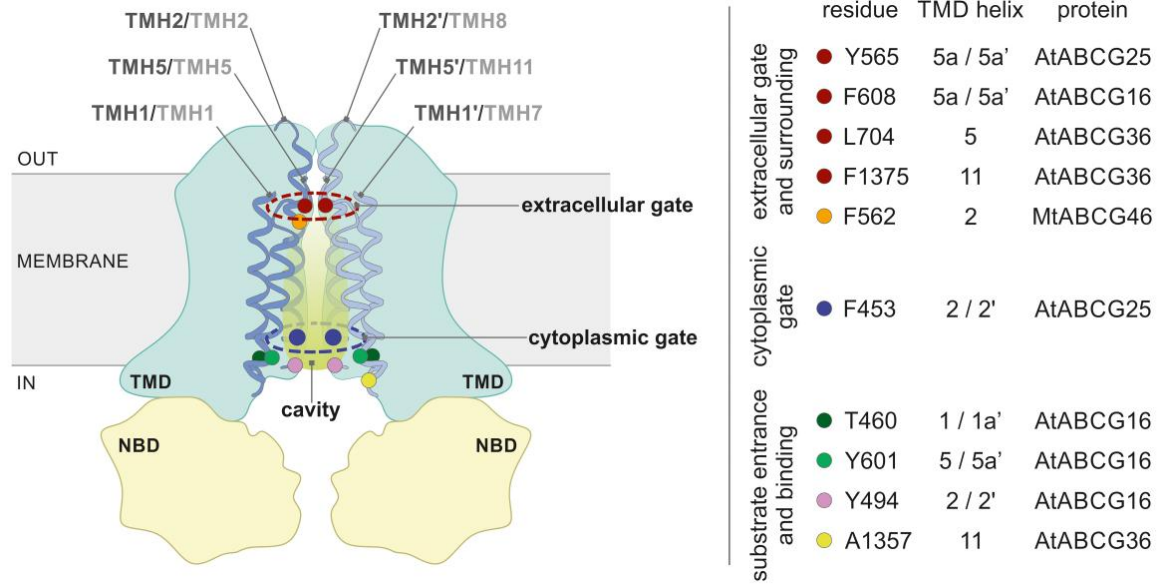
A schematic representation of the identified molecular determinants in plant ABCG transporters. NBDs and TMDs are tinted yellow and blue, respectively. Within TMDs, particular elements are highlighted: (1) helices forming the cavity from both halves are in dark blue and light blue, respectively. For the half-size transporters, helices are numbered in dark grey and numbered sequentially, with (‘) for the second half. For the full-size ABCG transporters, helices are numbered in light grey and the numbering preserves the order within a single polypeptide; (2) residues corresponding to substrate selectivity are indicated by colored dots and are listed on the right panel; and (3) regions corresponding to the extracellular (dark red circle) and cytoplasmic (dark blue circle) gates, respectively.

**Figure 3.**
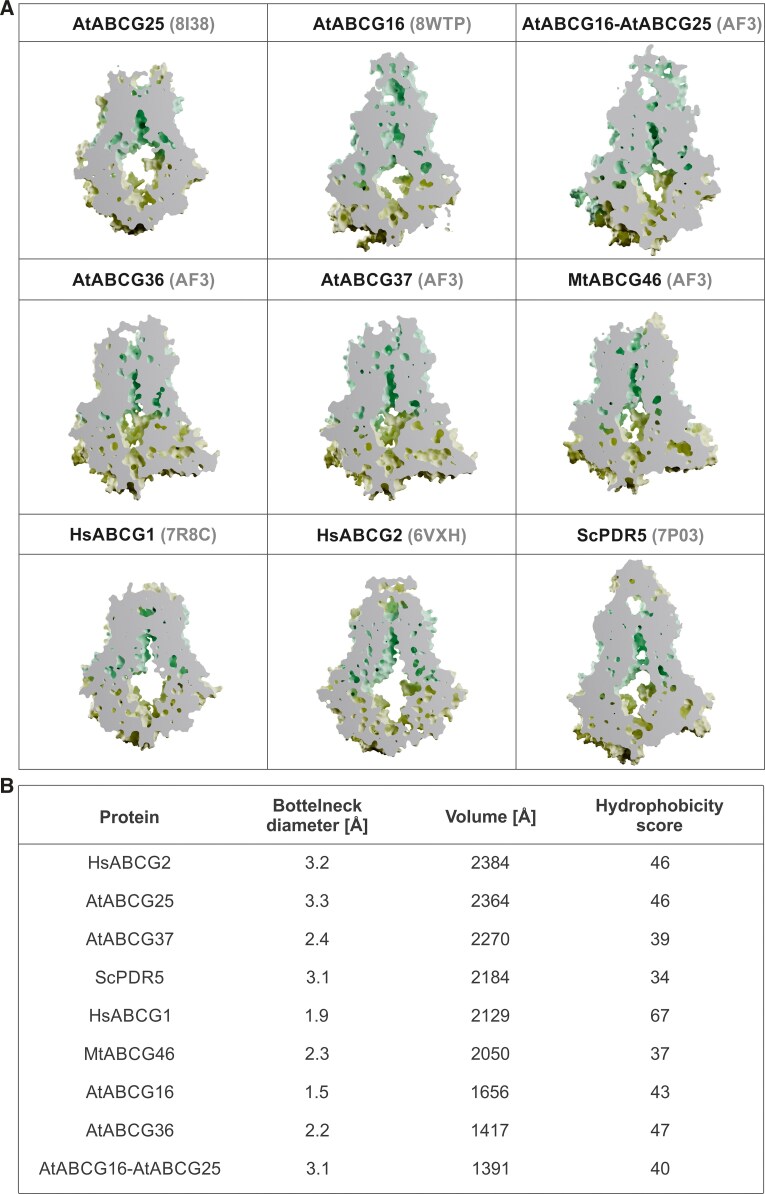
Comparative analysis of ABCG protein cavities. **A)** Cross-sections of ABCG transporters (AtABCG25, AtABCG16, AtABCG16-AtABCG25 heterodimer, AtABCG36, AtABCG37, MtABCG46, HsABCG1, HsABCG2, ScPDR5) show the architecture of cavities in their transmembrane regions (light green). The protein names are followed by PDB IDs for crystal structures or AF3 for AlphaFold3-predicted structures (Abramson et al. 2024). All structures are shown in their apo-state in the inward-facing open conformation except AtABCG16 (inward-facing closed). The crystal structure of HsABCG2 originally included imatinib, but the ligand was removed before cavity analyses. **B)** The accompanying table summarizes bottleneck diameters (calculated using CAVER 3.0; Chovancova et al. 2012), cavity volumes, and hydrophobicity scores (both calculated using mdpocket; Schmidtke et al. 2011). The bottleneck refers to the narrowest part of the access path leading to the central cavity. Hydrophobicity scores are calculated as the mean hydrophobicity of residues forming the cavity based on Monera et al. (1995) residue hydrophobicity scale, reaching values from −55 to 100 for polar aspartate and hydrophobic phenylalanine, respectively. ABCG transporters are ordered by volumes of their cavities.

Recently published structures and in silico modeling of ABCG transporters reveal a distinctive central cavity within 2 TMD domains, primarily formed by helices 2, 2′, 5, and 5′ in half-size ABCG proteins (Lee et al. 2016; Taylor et al. 2017; Huang et al. 2023; Ying et al. 2023; Xin et al. 2024) and corresponding helices 2, 5, 8, and 11 in full-size (Harris et al. 2021; Pakuła et al. 2023; Xia et al. 2024) (Fig. 2). Additionally, for AtABCG25 and AtABCG16, TMD helix 1 also was shown as being involved in cavity formation (Huang et al. 2023; Ying et al. 2023; An et al. 2024; Xin et al. 2024). Interestingly, the comparison of, for example, cavities observed in inward-facing yeast ScPDR5 structure and Medicago ABCG46 model revealed that in the latter, the cavity is small and disconnected, while in yeast, it is widely open (Pakuła et al. 2023). Such open cavities were also observed in human ABCG1 (Sun et al. 2021) and ABCG2 (Orlando and Liao 2020) (Fig. 3A). This raises the question about the possible impact of the cavity size and shape on the substrate binding/selectivity. Of note, for all structurally analyzed ABCG transporters, the translocated molecules were positioned, in the protein-molecule bound conformations, within the cavity region.

The complex and regulatory impact of the entire cavity for the translocation scenario in plant ABCGs is tempting but not surprising. Studies of human ABCG2 transporters identified a highly conserved “valve” structure in the central cavity, a feature shared among ABCG transporters in humans and fungi. The valve includes the di-leucine motif (L554 and L555, leucine plug), where the leucines from 2 halves face each other, thus forming a gate crucial for substrate translocation into the extracellular region cavity (Khunweeraphong et al. 2019). As mentioned above, structural analysis of AtABCG25 revealed the presence of 2 “gates” in the protein translocation pathway that controls the transport of ABA molecule: the “cytoplasmic gate” formed by F453 and the “apoplastic gate” formed by Y565 (Huang et al. 2023; Ying et al. 2023; Xin et al. 2024). The latter is a functional homolog of the HsABCG2 leucine plug, similarly as L704 together with F1375, which is crucial for AtABCG36 selectivity (Xia et al. 2024) or F608 in AtABCG16 that is controlling the closing and opening of the translocation pathway (An et al. 2024). Assuming the fact that the functioning of such a gate is independent of leucine conservation in the sequence, it was proposed to use the term “extracellular gate” instead of the leucine plug (Xia et al. 2024). Likewise, the F562 identified in Medicago ABCG46 corresponds to the F431 in HsABCG2. It was shown that F431 is highly conserved in human ABCG transporters and among ABCG2 homologs in several animal species. Intriguingly, this contrasts with plant full-size ABCGs, where, apart from F, other hydrophobic amino acids such as Y, L, and I occur at the corresponding position (Pakuła et al. 2023) (Fig. 2). However, the conservation context in animals and the role of F431 in transport remains to be discovered. The proposed role of the residues of the “extracellular gate” and its surroundings is to provide quality control contributing to substrate selectivity (Xia et al. 2024). In plants, it is supported by the described influence of substitutions within this region for AtABCG36 L704F (loss of camalexin transport) and L704Y (gain-of-IAA transport ability) and in MtABCG46 F562L (loss of *p*-coumaric transport) (Pakuła et al. 2023; Xia et al. 2024). Altogether, the evidence indicates that this particular region of the translocation path serves as a crucial site for molecular determinants that influence substrate selectivity.

A comparative analysis of mammalian ABC structures and prokaryotic counterparts demonstrates that eukaryotic ABCGs exhibit structural similarities to bacterial importers, such as ModBC and MalFGK_2_. However, they are radically different from the topological arrangement of the transmembrane helices. Moreover, the mentioned bacterial importers require additional binding proteins (ModA and MBP, respectively), which are crucial for the translocation of a limited number of molecules present in their environment (Ford and Beis 2019). In contrast, eukaryotic ABCG importers generally function without separate peripheral binding proteins for substrate capture. Instead, the substrate specificity and recognition in eukaryotic ABCG importers are typically mediated directly by the TMDs, which contain specialized regions or pockets that directly bind the substrate. In plants, the precise spatiotemporal presence of a transporter and the local source-sink concentration differences of translocated molecules can trigger the import and biological readout of these molecules. This can be illustrated by the Arabidopsis ABCG40, which is a plasma membrane ABA-uptake transporter present specifically in guard cells as well as seed embryos and is responsible for stomata closure and seed dormancy, respectively (Kang et al. 2010, 2015). Furthermore, it was proposed that a MacB-type ABC exporter seems a much more likely candidate for the originator of eukaryotic ABCG families than a ModBC-type importer (Ford and Beis 2019). However, the MacB-type ABC protein, which exports toxic compounds in an energy-driven manner, also collaborates with other partners—namely the MacA adaptor protein and TolC exit duct—to drive efflux out of the bacterial cell. The MacB TMD does not contain a central cavity for substrate passage but rather facilitates conformational changes across the membrane through a process called mechanotransmission (Crow et al. 2017).

It is worth noticing that plant ABC transporters may act in influx, efflux, or both at the same time. For instance, ABCB4/PGP4 was demonstrated as an influx (Terasaka et al. 2005), efflux (Cho et al. 2007), and both influx/efflux (Yang and Murphy 2009; Kubeš et al. 2012; Swarup and Péret 2012) carrier of auxin. The ABCB21 is a facultative auxin importer/exporter (Kamimoto et al. 2012). The previously mentioned ABCG40 has been shown to function as an influx carrier for ABA but an efflux carrier for cadmium (Lee et al. 2005; Kang et al. 2010). Similarly, the ABCG37 can function in both efflux (for 2,4-D and IBA) and influx (for cesium) (Růžička et al. 2010; Ashraf et al. 2021). As such, plant ABC transporters exhibit greater directional flexibility in transport compared with their animal counterparts. Molecular determinants behind this phenomenon remain to be discovered. Interestingly, mutation of specific residues within TMH 6 and 12 of human ABCB1/PGP resulted in loss of substrate efflux and altered the transport direction from efflux to uptake for some substrates, suggesting the presence of a regulatory switch that governs the direction of transport (Sajid et al. 2025).

**Figure 4.**
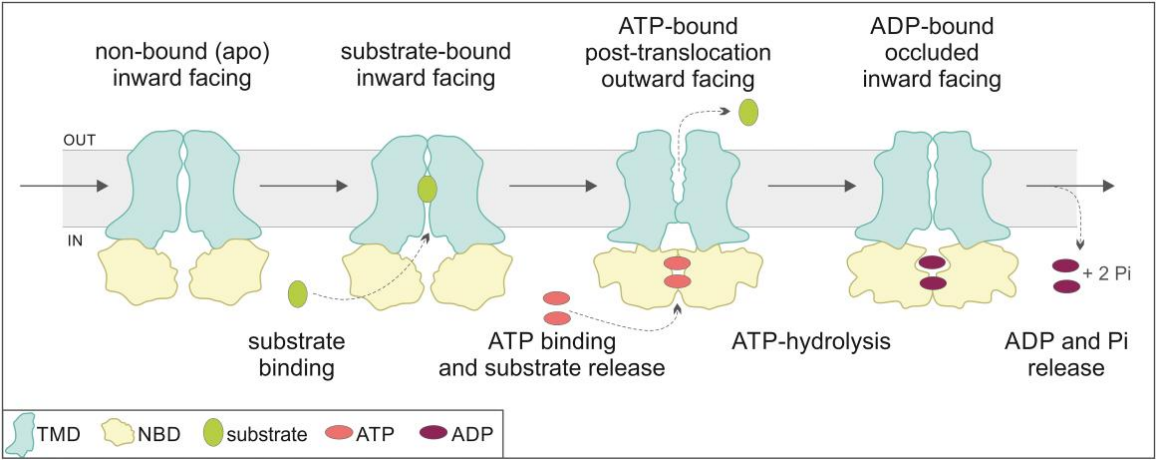
Simplified translocation cycle (export). In its nonbound (apo) state, the protein is in an inward-facing conformation, ready for substrate binding. From the intracellular side, the molecule enters the cavity formed by TMDs. Subsequently, the binding of ATP to the NBD causes the NBD to close together and opens the translocation pathway on the extracellular side, altering the protein's conformation to outward-facing and releasing the substrate. After ATP hydrolysis, the transporter returns to an inward-facing conformation with a translocation pathway closed on both sides. ADP release then triggers the transporter's reset to the apo state. This model is based on recent studies by An et al. (2024), which provide evidence for all 4 stages, including the occluded one, supported by specific structures obtained for AtABCG16.

The translocation mechanism can be briefly described in terms of alternating access involving inward-facing and outward-facing states, with the transitions between them coupled to the binding and hydrolysis of ATP (Thomas et al. 2020) (Fig. 4). Of note, structural analysis dedicated to the previously mentioned AtABCG16, by capturing protein structure in different nucleotide- and ligand-binding states, allowed for deeper insight into the substrate translocation process and complete the substrate translocation model (An et al. 2024). However, these data describe an exporter, and structural details concerning the import cycle of plant ABCG protein remain to be illustrated.

The ability of molecules to access the translocation path and reach the central cavity represents another factor that potentially influences substrate selectivity of plant ABCG proteins. As described above, plant exporter entry depends on certain crucial residues. For instance, in the AtABCG36, A1357 in TMD helix 11 is localized close toward the substrate entry side. A change of this amino acid results in losing the transport ability toward IBA as well as indole glucosinolate derivatives (Lu et al. 2015), possibly by narrowing the entrance to the pathway. Similarly, in AtABCG16 residues T460 (TMD helix 1) and Y601 (TMD helix 5a′), controlling the entrance to the substrate binding pockets were pointed to as critical for substrate recognition and translocation. Moreover, mutation of Y494 that is stabilizing the bound substrate impaired the AtABCG16 transport activity (An et al. 2024). Analyses of the above-mentioned structure of another exporter, AtABCG25, suggested that F453 may play a dual role along the translocation. First, as mentioned, by forming an entry gate, it controls the substrate entrance into the binding cavity. On the other hand, together with residues forming the central cavity, F453 might be involved in driving the substrate during translocation through the pathway. The latter is mainly constituted by TMD helices 2 and 5 from each half. The meaning of residues forming the interface between ABA and AtABCG25 was analyzed by alanine substitutions and transport assays. This revealed that residues forming the bottom part of the cavity (including among others Y565) are critical for ABA recognition and efflux (Ying et al. 2023) (Fig. 2). Similarly, the existence of additional filters impacting the entry to the cavity was investigated in the mentioned MtABCG46. The viability of paths that access the intracellular environment into the central cavity within TMDs was assessed for MtABCG46 substrates (*p*-coumaric acid and liquiritigenin) as well as chemically similar but not transported by this protein, isoliquiritigenin, and 7,4′-dihydroxyflavone. Calculations of energetic profiles of the binding energy between a molecule and protein along the path revealed that all tested molecules were able to reach the central cavity of the protein, albeit with much-reduced efficiency for isoliquiritigenin. However, only 2 of them (*p*-coumaric acid and liquiritigenin) were finally translocated to the other side of the membrane, as shown by the transport assays (Pakuła et al. 2023).

**Figure 5.**
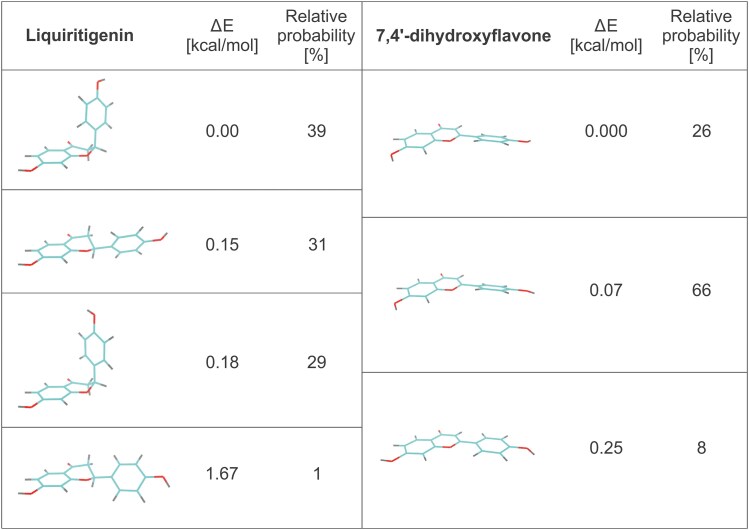
Conformational flexibility and spatial differences between MtABCG46 substrates. Structural differences between the transported substrate liquiritigenin and the nontransported substrate 7,4′-dihydroxyflavone are highlighted. Liquiritigenin adopts 4 distinct conformations while 7,4′-dihydroxyflavone has 3; their relative occurrence probabilities were calculated based on their relative energy (ΔE, kcal/mol) using CREST software (Pracht et al. 2024). Despite differing by only 1 bond, the conformational flexibility of these 2 molecules varies significantly. The conformers of liquiritigenin demonstrate greater diversity, with variations in the orientation of substituents. In contrast, 7,4′-dihydroxyflavone is more rigid and planar, with differences limited to hydroxyl group positioning and minor ring rotations. Coloring: carbon blue, oxygen red, hydrogen grey, structural formulas of both molecules are available in Fig. 1.

It is worth noting that the physicochemical properties of transported molecules may affect their interaction with the transporter (Fig. 1). Molecular hydrophobicity governs the kinetics of movement across biological membranes, which is one of the basic mechanisms of cellular transport (Buchanan et al. 2015). However, hydrophobic interactions play a role also in interactions between low-molecular-weight compounds (such as phytohormones and specialized metabolites) and various transporters (Nedvěd et al. 2021). The impact of hydrophobicity is visible in, for example, the amino acid composition of binding cavities of ABCG proteins along with their substrates. For instance, the HsABCG1, which transports cholesterol, possesses the most hydrophobic cavity, whereas the MtABCG46, which translocates phenylpropanoids, exhibits significantly lower hydrophobicity (Sun et al. 2021; Pakuła et al. 2023) (Fig. 3B). Furthermore, steric effects, although they are nonbonded interactions, influence the shape and behavior of molecules, as shown for ABA isoforms and their binding by AtABCG25. In silico positioning of the (S)-(+)-ABA molecule within AtABCG25 protein shows that it fits well in the substrate binding site (accordingly with observed densities in the cavity), while positioning of nontransported (R)-(−)-ABA revealed the steric conflict of the dimethyl ABA group with the side chain of T552 (Huang et al. 2023). Similarly, the IAA molecule, while bound into the AtABCG36, is losing internal molecular stability in contrast to substrates such as IBA and camalexin that remain intact (Xia et al. 2024). Of note, all of the molecules translocated by AtABCG36 have an indole core, which may indicate the bias of this transporter toward indole compounds, while the substituents on the aliphatic chain and the physicochemical properties of the latter may determine the translocation fate (Fig. 1). In the case of MtABCG46, one of the primary factors may be the difference in the bonding of the central rings. Liquiritigenin contains a chroman-4-one ring, while 7,4′-dihydroxyflavone features a chromone ring with conjugated bonds. This conjugation in the chromone creates a rigid, planar structure and limits the rotation of the phenolic group. In contrast, the nonconjugated chroman-4-one ring in liquiritigenin allows for greater conformational flexibility and movement of the phenolic substituent between axial and equatorial positions (Fig. 5).

The membrane environment itself and the influence of sterols represent additional regulatory factors affecting ABCG protein activity. This was shown for HsABCG2, for example, where protein activity requires cholesterol (Hegedüs et al. 2015; Jackson et al. 2018). However, it remains unclear whether this effect results from changes in lipid bilayer structure or from sterols acting as allosteric modulators through specific binding to ABCG2 (Nagy et al. 2021). Plant cells lack cholesterol, but fitosterols may serve the same purpose, and an argument for this may be the observation of 2 sterol densities within the AtABCG25 cavity in an inward-facing conformation (Ying et al. 2023; Xin et al. 2024). Moreover, it was shown that the presence of sterols during the purification and/or reconstitution significantly increased the ATPase activity of AtABCG25 (Huang et al. 2023; Ying et al. 2023; Xin et al. 2024) as well as AtABCG16 (An et al. 2024), thus indicating the regulatory role of sterols also for plant ABC transporters and emphasizing the meaning of the protein environment. It is an additional argument for rational choice of a system for their heterologous expression, purification as well as transport experiments. For more details, see (Lefèvre and Boutry 2018).

Previous work on posttranslational modifications of ABC transporters has shown that they may influence overall protein folding, membrane trafficking, as well as activity and substrate preferences. For instance, mutation of 2 glycosylation sites (N746 and N754) in mammalian ABCC4 (MRP4) causes substrate-selective effects on the transport of prostaglandin E2 but not estradiol glucuronide (Miah et al. 2016). Also, the activity of 2 Arabidopsis ABCB proteins, ABCB1 and ABCB19, was shown to be controlled by kinases PHOTOTROPIN1 and PINOID, respectively (Christie et al. 2011; Henrichs et al. 2012). However, there are only a few reports concerning the posttranslational regulation of plant ABCG proteins and, in particular, how it might affect substrate specificity. The camalexin secretion by AtABCG34 (PDR6) has been modulated by the activity of 2 AGC kinases OXI1 and AGC2 phosphorylating 7 threonine and serine sites (Han et al. 2024). Also, recently published research data on AtABCG36 revealed that LRR receptor kinase QSK1 was shown to coordinate transporter substrate preferences by phosphorylating residues within the linker S825 and S844 and thus repressing IBA export and promoting camalexin export (Aryal et al. 2023). It is worth emphasizing that transporters do not act independently but in a network of complex molecular interactions; thus, uncovering their interactomes is desirable because it could provide us with detailed spatiotemporal information on ABC transporter activity (for review, see Geisler et al. 2017).

The ABCG subfamily represents the largest ABC subfamily in land plants, comprising approximately 40% of all genome-encoded ABC proteins, with nearly equal distribution between full- and half-size ABCGs (Banasiak and Jasiński 2022). From an evolutionary perspective, half- and full-size transporters have a common ancestor, and full-size could derive from gene duplication of a half-size transporter gene (Gräfe and Schmitt 2021). Full-size ABCGs are characteristic of plants, fungi, oomycetes, brown algae, and slime molds (for review, see Kang et al. 2011; Do et al. 2021), and it is hypothesized that this duplication occurred before the evolutionary separation of plants and fungi, followed by the deviant evolution of the full-size ABCG transporters in the respective kingdom (Crouzet et al. 2006). Importantly, half-size ABCGs may form dimers with multiple partners and thus can form various functional units representing a driving force behind the adaptation of this versatile transport system to many structurally diverse molecules and biological scenarios. Dimerization with different partners allows not only for forming alternative binding sites, thus affecting substrate selectivity, but also may influence also the localization of the functional transporter. For instance, both AtABCG11 and AtABCG12 are involved in the transport of cuticular wax precursors but exhibit different behavior. AtABCG11 can form homodimers that localize to the plasma membrane. While AtABCG12 trafficking to the plasma membrane depends on heterodimerization with AtABCG11, otherwise AtABCG12 is not able to exit the ER and thus form inclusions (McFarlane et al. 2010). The expression pattern of ABCG11 extends beyond that of ABCG12, including regions such as emerging lateral roots where cutin and waxes are not synthesized (Panikashvili et al. 2007), suggesting additional dimerizing partners and potential functions. Indeed, AtABCG11 was shown to participate in vascular system development together with AtABCG9 and AtABCG14. While AtABCG9 was able to form homodimers as well as dimerize with AtABCG11, AtABCG14 forms an obligate heterodimer with ABCG11 (Le Hir et al. 2013). The abovementioned interaction analyses were mainly based on biomolecular complementation assay and/or co-immunoprecipitation, and the postulated roles of heterodimers were raised mainly on phenotypic observations. Intriguingly, further studies on AtABCG14's role in cytokinin transport revealed that AtABCG14 can form homodimers, which was additionally supported by complex transport assays conducted in homologous (Arabidopsis cells) and heterologous (tobacco BY2 and yeast cells) systems (Zhao et al. 2023). Another interesting example is AtABCG16, exhibiting dual localization in the plasma membrane and nuclear envelope, where it provides cellular efflux of JA and nuclear entry of jasmonyl-isoleucine. Based on distinct substrate specificity and affinity, AtABCG16 likely forms homodimers in the nuclear envelope while forming heterodimers with other partners in the plasma membrane, where additional ABCG JA transporters are present (Li et al. 2017). Recently published research suggested that AtABCG16 may form a heterodimer together with AtABCG25, resulting in alternate localization and substrate selectivity (Zhou et al. 2024). It is postulated that heterodimer AtABCG16-AtABCG25 controls the entry of ABA-glucosyl ester into the ER membrane interface; however, this study is lacking in the demonstration of ABA-GE direct transport assay performed by this heterodimer. These findings highlight the need for developing reliable and efficient systems to study heterodimer transport activity.

Another important aspect of heterodimer formation is structural circumstances such as spatial adjustment and the possibility of interaction between particular amino acid residues from dimerizing proteins. Proper formation of certain elements such as transmembrane cavity, for example, assures the functioning of the transporter. In the case of the only structurally analyzed ABCG heterodimer, human ABCG5/ABCG8 (Lee et al. 2016), the central cavity is formed by helices 2 and 5. However, as mentioned earlier, in AtABCG16 and AtABCG25 helix 1 also is involved in cavity formation. These structural elements may play a crucial role in determining heterodimerization specificity. Furthermore, the amino acid composition of extracellular and cytoplasmic gate regions plays an important role in forming heterodimers and consequently in the translocation of specific molecules. The AtABCG16/AtABCG25 heterodimer is predicted by AF3 to retain a similar composition in helices 2, 5, and 1, where the same residues are involved in the formation of the extracellular gate. However, the cytoplasmic entrance to the cavity formed by the heterodimer appears to adopt an intermediate architecture, being wider than in ABCG16 but narrower than in ABCG25 (Fig. 3B), which might facilitate the transport of spatially larger substrates by the heterodimer. There is also a strong constriction in the center of the heterodimer's cavity likely responsible for its bifurcation similar to AtABCG16 (Fig. 3A). Nonetheless, the relationships between this amino acid diversity, resulting structural configurations, and substrate specificity and transport efficiency remain an open question warranting further investigation.

## Conclusions

Elucidation of molecular determinants behind the translocation process fulfilled by plant ABC transporters is an important part of their functional characterization. Recent discoveries highlight the phenomenon of multispecificity and raise questions about its physiological significance, particularly in contexts such as specialized metabolite biosynthesis and phytohormone interactions (see Outstanding Questions). For example, in legumes, the MtABCG46 governs the carbon flow between the phenylpropanoid branches dedicated to symbiotic interaction and defense, respectively (Biała and Jasiński 2018; Pakuła et al. 2023). Likewise, AtABCB19 coordinates the distribution of 2 important phytohormones: auxin (Blakeslee et al. 2007; Bailly et al. 2014) and brassinosteroids (Ying et al. 2024). Finally, AtABCG36 uncouples the growth phytohormones signaling from the transport of defense-specialized metabolites (Aryal et al. 2023; Xia et al. 2024). The latter case brings our attention to the future research perspective, which is deciphering the regulatory context of various fluxes. The AtABCG36 work has shown that phosphorylation and action of receptor kinases may represent a direct regulatory circuit allowing to control transporter substrate preference during various biological scenarios (Aryal et al. 2023). However, an intriguing intrinsic regulatory context also exists, including the entrance of substrate into the binding pocket and their recognition, which is regulated by particular residues that constitute sort of initial filters as revealed for AtABCG36 (Lu et al. 2015) or AtABCG16 (An et al. 2024). Moreover, studies of the latter reveal that ABC transporters can possess 2 independent substrate entrances leading to separate binding pockets connected to a shared cavity (An et al. 2024), challenging the previous model of a single, straightforward translocation pathway within TMDs.

Farther on, residues determining the transport through the translocation pathway such as the “cytoplasmic gate” and providing quality control “extracellular gate” were identified, and their crucial role in substrate selectivity was demonstrated. Attention was recently brought to the fact that the fusion of all 4 domains in eukaryotic ABC transporters via linkers may be an adaptation to the increased transport complexity. Moreover, linker length appears to be important, and as such the presence of the linker raises the possibility of volume exclusion effects constraining the conformational landscape of the linker region (Ford et al. 2020).

Finally, structural data provide new perspectives on our existing understanding of these transporters. For instance, the AtABCB19 has long been implicated in auxin transport. Interestingly, Ying et al. (2024) identified an alternative role for ABCB19 as a transporter of brassinosteroids. By combining the cryo-EM structural studies, analysis of binding affinity, and transport assays together with genetic analyses, the role of ABCB19 and its close homolog, ABCB1, in brassinosteroid signaling was demonstrated, thus providing a comprehensive understanding of the ABCB19 role in plant biology. Furthermore, structural analysis of ABCG proteins, which are involved in the distribution of phytohormones and specialized metabolites, such as AtABCG25 (Huang et al. 2023; Ying et al. 2023; Xin et al. 2024), AtABCG16 (An et al. 2024), AtABCG36 (Xia et al. 2024), or MtABCG46 (Pakuła et al. 2023), provided deeper insights into the multispecificity that greatly supplement biological relevance of those transporters. The acquired knowledge will not only aid in resolving the mechanisms underlying ABC-driven transport but will be beneficial for contemporary agriculture. The ABC-targeted research strategies can be applied in biotechnology for engineering of biosynthetic pathways and production of metabolites, for example, as well as in agriculture for breeding and the development of new varieties possessing desirable traits.

**ADVANCES BOX**

- The multispecificity of a single ABC transporter could be recognized as a regulatory element in plant physiology.
- The presence of numerous access pathways within a single transporter may modulate the activity of multispecific proteins.
- Multiple selective filters regulate the selective translocation of molecules at distinct levels or stages. These filters include entrance-controlling residues, a cytoplasmic gate, and an extracellular gate.
- Structural analyses capturing several bound statuses of ABCG transporters allowed us to elucidate the transmembrane translocation cycle.
- Structural analyses, along with functional characterization and transport experiments, are essential for comprehending the physiological significance of multispecificity.

**OUTSTANDING QUESTIONS BOX**

- How widespread is multispecificity among plant ABC transporters?
- How do intrinsic and exogenous factors regulate multispecific transport?
- Do multiple binding sites exist in plant ABCG proteins? How is access to them modulated, and do binding sites vary in substrate affinity?
- Do the full-size ABCG transporters represent a refined evolutionary solution for the controlled efflux of endogenous molecules?

## Data Availability

The data underlying this article are available in the manuscript.

## Dive Curated Terms

The following phenotypic, genotypic, and functional terms are of significance to the work described in this paper:

ABCB19 Gramene: AT3G28860
ABCB19 Araport: AT3G28860

ABCB1 Gramene: AT2G36910
ABCB1 Araport: AT2G36910

pDR5 Gramene: AT2G37280
pDR5 Araport: AT2G37280

PDR6 Gramene: AT2G36380
PDR6 Araport: AT2G36380

ATP CHEBI: CHEBI:15422

OXI1 Gramene: AT3G25250
OXI1 Araport: AT3G25250

PINOID Gramene: AT2G34650
PINOID Araport: AT2G34650

QSK1 Gramene: AT3G02880
QSK1 Araport: AT3G02880

ABCG46 Gramene: Medtr_2g102670
ABCG46 Araport: Medtr_2g102670
